# Left bundle branch block in serious hyperkalaemia: rate-dependency?

**DOI:** 10.1007/s12471-016-0865-z

**Published:** 2016-07-25

**Authors:** R. Joustra, M. Boulaksil, H. W. Meijburg, M. C. Daniëls

**Affiliations:** 1 Department of Cardiology, Jeroen Bosch Hospital, ’s-Hertogenbosch, The Netherlands; 2 Department of Cardiology, Radboud University Medical Center, Nijmegen, The Netherlands

Dear Editor,

Fransen et al. [1] published a Heart Beat in this journal showing a case with rate-dependent left bundle branch block (LBBB) in a patient with serious hyperkalaemia. Critical appraisal of the ECGs, however, suggests that the hyperkalaemia by itself may be the explanatory mechanism of the LBBB rather than the increased heart rate. As shown in Fig. 1, the QRS complex immediately following a prolonged RR interval (10^th^ QRS complex) is still widened, ruling out rate dependency as the underlying cause of left bundle branch aberrancy in this case. Possibly, there was a time interval between the two ECGs explaining the occurrence of LBBB here. If the second ECG was performed after initiation of therapy, a decrease in the potassium level rather than the heart rate would be more explanatory.
